# Metaverse enabled virtual reality and simulation training for medical education review

**DOI:** 10.1016/j.isci.2026.115656

**Published:** 2026-04-13

**Authors:** Zhisheng Chen

**Affiliations:** 1 Wuxi Taihu University, Wuxi, China

**Keywords:** Education in health technology, Nursing education

## Abstract

The integration of metaverse technologies into medical education has attracted increasing attention in recent years. This study conducts a scoping review following PRISMA-ScR guidelines to examine the applications, benefits, and challenges of metaverse-enabled virtual reality and simulation training in medical education. A systematic search was performed across Web of Science, Scopus, and PubMed databases for studies published between 2020 and 2025. The review synthesizes existing evidence regarding immersive learning environments, simulated patient training, collaborative learning, and cross-geographical education. Additionally, technical, ethical, and social challenges associated with metaverse adoption in medical education are discussed. The findings highlight the potential of metaverse technologies to enhance experiential learning, clinical skill development, and global collaboration in medical training while identifying key directions for future research.

## Graphical abstract

**Figure:**
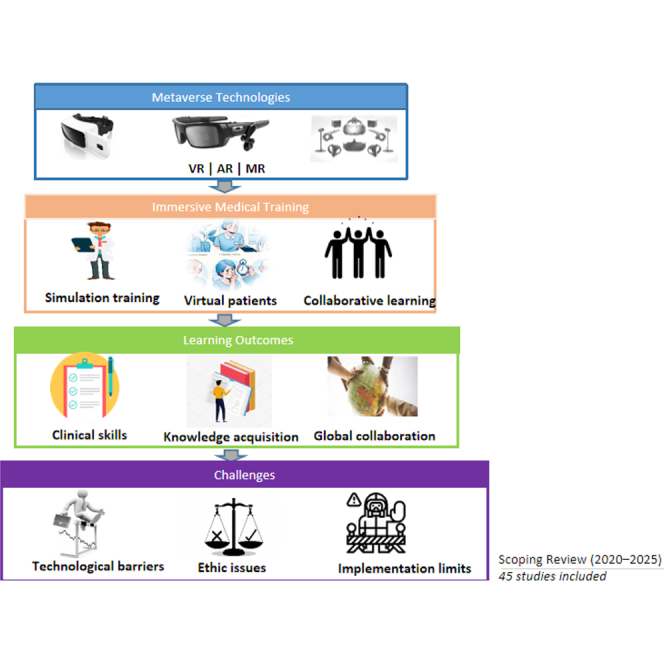

## Introduction

Medical education plays a pivotal role in the training and development of medical professionals, yet it faces numerous challenges and limitations.^1^ The conventional model of medical education heavily relies on physical resources such as laboratories, clinical internships, and real-life clinical scenarios. However, the availability and accessibility of these resources are often restricted by temporal and spatial constraints.^2^ Additionally, fostering interdisciplinary collaboration and embracing distance learning are essential for nurturing well-rounded medical professionals.^3^ Consequently, there is an urgent imperative to explore novel pedagogical approaches and harness technological advancements to address the evolving needs of medical education.^4^

The emergence of the metaverse—a virtual digital ecosystem integrating virtual, augmented, and mixed reality (MR)—offers new possibilities for immersive and collaborative learning in medical education.^5^ Within such environments, students can engage in realistic and safe simulations to enhance their clinical reasoning and procedural skills.^6^

Prior research across disciplines demonstrates that metaverse technologies can enhance interaction, collaboration, and engagement among learners and educators.^7^^,^^8^ Such findings provide a foundation for examining their pedagogical value within medical training contexts.

In addition to its collaborative potential, the metaverse enables personalization and global sharing of educational resources, allowing learners to tailor experiences to their needs and access high-quality instruction beyond geographical boundaries.

Therefore, the primary objective of this study is to comprehensively examine the potential and significance of utilizing metaverse applications in the field of medical education. By conducting the analysis of existing literature, we aim to evaluate the advantages and possibilities of metaverse technology in delivering immersive learning experiences, addressing practical and experimental limitations, fostering interdisciplinary collaboration and distance education, facilitating personalized learning, and promoting the sharing of educational resources. Additionally, we will explore the challenges and limitations that may arise in the implementation of metaverse technology in medical education, and provide relevant recommendations to promote its application.

While several recent reviews have examined the application of virtual and augmented reality (AR) in medical education, few have systematically mapped these technologies within a unified metaverse framework. This study advances prior work by integrating a taxonomy-based conceptual distinction among VR, AR, MR, and the metaverse, as visualized in Figure 2, and by applying the PRISMA-ScR methodology to synthesize evidence across these domains. Furthermore, by introducing a structured evidence table that assesses metaverse features, learning outcomes, and study quality, this paper bridges the gap between conceptual discussions and empirical evaluation. These methodological and analytical innovations distinguish the present review from existing literature and justify its contribution to the field.

The significance of this study lies in its exploration of the prospects of metaverse technology in medical education, serving as a theoretical and practical foundation for the enhancement and innovation of medical education. By highlighting the advantages and potential of metaverse technology in medical education, this study offers guidance and decision-making support to educational institutions and medical educators. Educational institutions can learn from successful cases of metaverse technology applications to optimize teaching methods and resource allocation, thereby improving the quality of instruction. Medical educators can gain insights into the characteristics and application modes of metaverse technology, enabling them to select suitable technological tools that align with their teaching needs, ultimately enhancing teaching effectiveness and student satisfaction.

The structure of this study is organized as follows: the methods are presented first, followed by a literature review on metaverse technologies and their relevance to medical education; subsequent sections discuss benefits, implementation challenges, and representative cases; the paper concludes with future prospects and the overall significance of integrating metaverse technology into medical education.

## Methods

### Search strategy

This study adopted a scoping review approach following the PRISMA-ScR guidelines to map existing research on metaverse-enabled simulation and virtual training in medical education. A comprehensive literature search was conducted in the Scopus, Web of Science, and PubMed databases.

### Search terms and time frame

The search included publications from 2020 to 2025, using combinations of the keywords: “metaverse,” “virtual reality (VR),” “AR,” “MR,” “simulation training,” and “medical education.”

### Inclusion and exclusion criteria

Studies were included if they: (1) addressed the application of metaverse, VR, AR, or MR technologies in medical education or training; (2) were peer-reviewed journal articles; and (3) provided empirical, conceptual, or review-based insights. Excluded were: (1) non-English papers, (2) conference abstracts without full texts, and (3) studies focusing on non-medical education contexts.

### Screening and selection

**Figure 1:**
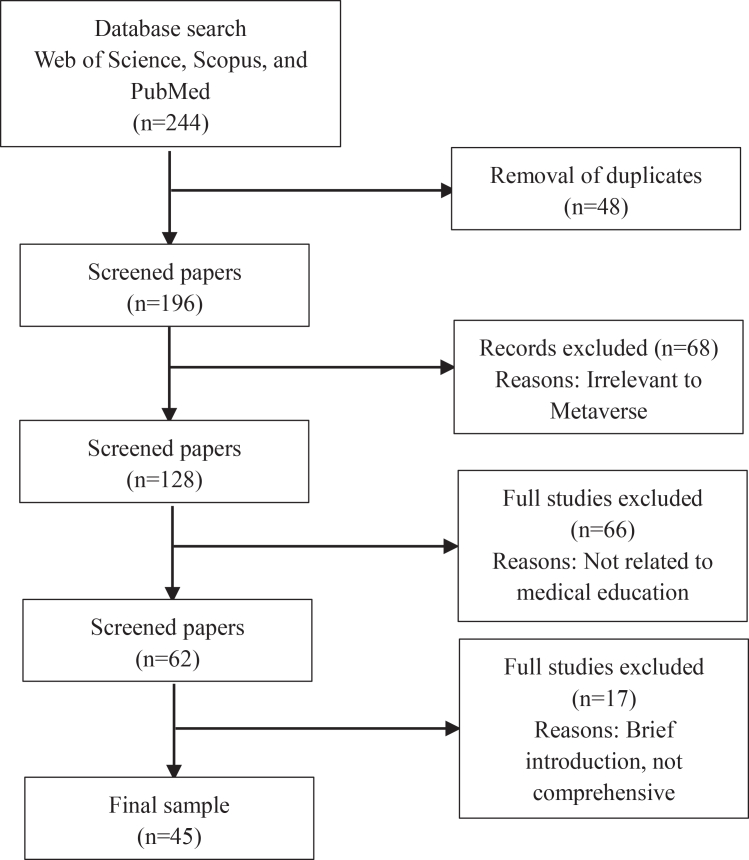
PRISMA flow diagram

A total of 244 records were identified through Web of Science, Scopus, and PubMed. After removing 48 duplicates, 196 papers remained for title and abstract screening. Sixty-eight records were excluded at this stage for being irrelevant to the metaverse. The remaining 128 full-text papers were assessed for eligibility, and 66 were excluded as they did not relate to medical education. Another 17 were excluded due to being brief introductions or lacking sufficient methodological or analytical depth. Consequently, 45 studies met the inclusion criteria and were included in the final synthesis. The selection process is illustrated in Figure 1.

### Data extraction and synthesis

Each selected paper was coded according to author, year, study design, sample, technology type, outcomes, and key findings. The results were thematically synthesized into three analytical dimensions: technological, pedagogical, and ethical governance.

Figure 1 illustrates the literature identification, screening, eligibility, and inclusion process following PRISMA-ScR guidelines.

## Literature review

### Metaverse technologies and their relevance to medical education

The metaverse, encompassing VR, AR, and artificial intelligence technologies, offers significant potential for medical education.^9^ In this study, the metaverse is operationally defined as a shared, persistent, and interoperable three-dimensional virtual environment that enables multi-user interaction and continuity of identity and data across settings. While VR, AR, and MR provide immersive experiences, the metaverse extends these technologies by integrating them within a networked ecosystem that supports synchronous participation and long-term persistence. Accordingly, throughout this review, only studies describing multi-user or persistent virtual learning environments were classified as metaverse-based, whereas single-user VR or AR applications were treated as immersive technology tools rather than full metaverse contexts.

**Figure 2:**
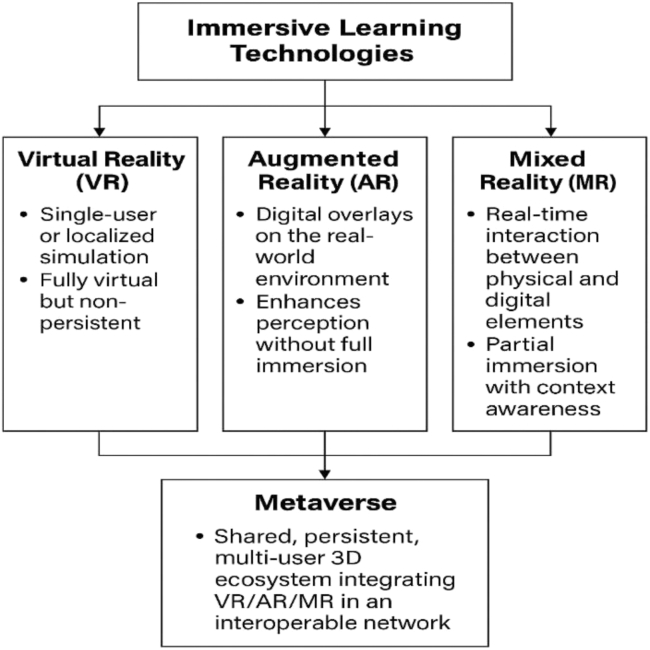
Conceptual taxonomy of immersive technologies and the metaverse ***Note***: The metaverse extends beyond isolated VR/AR/MR applications by emphasizing persistence, interoperability, and multi-user social interaction.

To ensure conceptual clarity, Figure 2 visually distinguishes immersive technologies of VR, AR, and MR from the metaverse, emphasizing that the latter integrates these components into a shared, persistent, and multi-user 3D ecosystem.

In this section, we explore the technological aspects of the metaverse and its relevance to medical education based on existing research.

Firstly, the use of metaverse technology in medical education provides students with realistic and immersive learning experiences.^10^ Through VR simulations, students can engage in hands-on training within simulated medical scenarios, such as operating rooms or emergency rooms.^11^ This enables them to interact with virtual patients, enhancing clinical skills and decision-making abilities.^12^

Augmented reality technology also holds promise for medical education. By using AR devices, such as AR glasses, students can integrate virtual information with the real world.^13^

Furthermore, the integration of artificial intelligence technology is crucial in medical education. AI can analyze students’ learning data and behaviors to provide personalized learning advice and feedback based on individual needs and proficiency levels.^14^ This personalized approach helps students better grasp medical knowledge and improve learning outcomes.^15^^,^^16^

Moreover, metaverse technology enables data analysis and visualization techniques that allow medical educators and students to monitor learning progress and performance.^7^ By collecting and analyzing large amounts of learning data, educators can assess students’ progress and tailor instruction accordingly. Students can visualize their learning progress, fostering self-reflection and enabling adjustment of learning strategies.

Building on these technological foundations, the next subsection outlines the defining characteristics of the metaverse and their educational relevance.

### Metaverse characteristics and its relevance to medical education

Building on these technological components, the metaverse can be characterized by four key attributes (virtuality, diversity, interactivity, and sharing), each of which carries specific educational implications.

First, the metaverse creates a VR by employing computer-generated images and sounds, offering users an immersive experience within various scenarios and activities, and allowing them to engage with objects and characters in the virtual environment.^17^^,^^18^

Second, the metaverse exhibits diversity by simulating a wide range of scenes and environments, including cities, forests, oceans, and more. Additionally, it provides diverse experiences and activities, such as games, social interactions, and learning opportunities, catering to the diverse needs and interests of users.^19^

Third, the metaverse promotes interactivity as users can actively interact with objects and characters in real time through VR devices or other interactive technologies.

Finally, the shared nature of the metaverse further contributes to its relevance in medical education. Users have the ability to engage in real-time social interactions and collaborate with others.^20^

In medical education, these technological characteristics collectively enhance experiential learning, knowledge retention, and collaboration. They allow students to practice clinical decision-making safely, access diverse learning materials, and engage in authentic team-based simulations. By synergizing metaverse technologies with the specific needs of medical education, innovative and effective teaching models can be developed to improve training quality and learner engagement.^5^^,^^21^^,^^22^

## Benefits and opportunities

### Simulated patient training

Simulated patient training, a crucial learning method in medical education, can be enhanced through the utilization of the metaverse.^23^^,^^24^^,^^25^ By harnessing VR technology, we can create realistic virtual patients, enabling students to engage in hands-on training within a virtual environment.^26^ This immersive experience offers several benefits and opportunities for students:

Firstly, simulated patient training facilitates the development of effective communication skills with patients, a vital competency for physicians.^27^^,^^28^ Through interactions with virtual patients, students can practice asking relevant questions, attentively listening to patients, and addressing their emotional needs. This practical training helps nurture students’ communication skills and interpersonal abilities.^29^

Secondly, simulated patient training enhances students’ proficiency in history taking, a fundamental aspect of medical practice.^26^^,^^30^^,^^31^ Accurately gathering a patient’s medical history is essential for accurate diagnosis and treatment planning. By engaging in simulated conversations with virtual patients, students can learn how to ask pertinent questions, gather essential information, and analyze and interpret the collected data. This hands-on training aids in the development of comprehensive history-taking skills.

Lastly, simulated patient training strengthens students’ competence in performing physical examinations.^32^^,^^33^^,^^34^ Physical examination plays a crucial role in assessing a patient’s physical condition. Through interactions with virtual patients, students can acquire proper physical examination techniques such as palpation, auscultation, and observation. This practical training helps refine students’ accuracy and sensitivity in conducting physical examinations, ultimately enhancing their clinical practice.

By leveraging the metaverse for simulated patient training, medical education can provide students with immersive and realistic learning experiences, fostering the cultivation of essential skills and competencies necessary for their future medical careers.

### Effects and applications of simulation training

The integration of simulation training in the metaverse has profound effects and diverse applications in medical education. Through realistic medical scenarios and cases, students can engage in repetitive practice sessions without the constraints of time and space, enabling them to enhance their diagnostic and therapeutic skills.^2^^,^^6^^,^^35^

Simulation training is applicable across various medical specializations. In the field of surgery, students can gain familiarity with surgical instruments and techniques by participating in surgical simulations within the metaverse. This enables them to practice different surgical steps, manage risks, and address complications, thereby improving their surgical proficiency and reducing errors.^36^

Similarly, in emergency medicine, students can develop their ability to recognize and manage acute and critical illnesses through first aid simulations in the metaverse. They can confront diverse emergency situations, such as cardiac arrest and severe trauma, while honing their emergency response and teamwork skills. This prepares them for real-world emergency care scenarios.^37^^,^^38^

Simulation training also holds great potential for medical teaching and assessment. In the metaverse, educators can create virtual scenarios and cases to guide students through simulation training and facilitate case discussions. By observing students’ performance and decision-making processes, teachers can provide real-time feedback and guidance, fostering continuous improvement and personalized learning. This approach nurtures independent learning and professional development.^2^^,^^39^

### Facilitation of collaborative learning

Collaborative learning is a crucial component of medical education, and the metaverse platform facilitates this process by enabling students to engage in collaborative problem-solving, case discussions, and knowledge sharing with peers, faculty, and professionals. This collaborative learning environment fosters interdisciplinary cooperation and teamwork skills.

Within the metaverse, students can actively participate in multi-person virtual meetings and team projects, collaborating with fellow students from diverse disciplinary backgrounds to address complex medical problems.^40^ Through open discussions and collaboration, students can exchange perspectives and experiences, leveraging each other’s expertise to gain a comprehensive understanding.

Real-time communication and interaction are facilitated through the metaverse platform, allowing students to engage in voice or chat-based discussions.^22^^,^^41^ Additionally, the platform provides tools such as shared documents to facilitate information sharing and collaborative editing among students. This real-time collaborative learning environment enhances cooperation, nurturing students’ teamwork skills.

Collaborative learning enables students to benefit from diverse perspectives and professional backgrounds.^5^^,^^10^ By collaborating with students and professionals from around the world, leveraging the cross-regional nature of the metaverse platform, students gain international exposure that broadens their horizons and cultivates cross-cultural communication and collaboration skills.

Moreover, collaborative learning not only enhances students’ individual learning and growth but also develops their teamwork and leadership abilities.^7^ In team projects, students learn to coordinate tasks, allocate resources effectively, and work together to solve problems. This teamwork experience is invaluable for their future medical practice and career development.

### Cross-geographical learning

Cross-geographical learning is a significant advantage offered by the metaverse platform, enabling students to engage with medical education resources from various locations worldwide.^40^ Through VR, students actively participate in international conferences and virtually explore laboratories of different medical schools within the metaverse. This immersive experience expands their perspectives and provides a globalized medical education encounter.

Traditionally, geographic constraints have hindered students’ access to global medical resources. However, the advent of the Metaverse overcomes this limitation. Students can now interact in real-time with medical experts and peers from around the globe through the metaverse platform, facilitating knowledge sharing, experiential learning, and the exchange of the latest medical research findings.^10^ This cross-geographical learning opportunity not only broadens students’ academic horizons but also fosters communication and collaboration within the international medical community.

Participating in international conferences allows students to personally engage with medical experts from diverse countries and cultural backgrounds.^5^ They present their research findings, while also gaining valuable insights into global medical advancements and trends. This cross-cultural exchange cultivates students’ ability to collaborate across disciplines and global perspectives, preparing them for future medical practice.

Furthermore, visiting virtual laboratories at different medical schools enables students to access medical education resources from various regions.^42^^,^^43^^,^^44^ They observe different teaching models and practical experiences, thereby gaining insights into medical education in different areas. This cross-regional learning experience stimulates students’ curiosity, inspires critical thinking about medical education, and promotes personal growth and development.

### Data analysis and research

Data analysis and research play a crucial role in enhancing medical education within the Metaverse platform.^5^^,^^45^ Through the collection and analysis of students’ behavioral data and learning outcomes, we can evaluate the effectiveness of teaching and learning, providing valuable insights for educational reforms. Additionally, researchers can leverage the metaverse platform to conduct medical education research, exploring innovative teaching strategies.

Data analysis enables us to assess teaching effectiveness and optimize instructional strategies.^37^ The metaverse platform records students’ behavior processes in virtual medical scenarios, as well as their learning outcomes. By analyzing this data, we gain a deeper understanding of student learning performance, allowing us to evaluate the effectiveness of instruction and identify areas for improvement. For instance, we can analyze student performance in simulated surgeries, assessing their operative skills, accuracy in diagnosing cases, and teamwork abilities. These data analyses provide targeted feedback to teachers, enabling them to refine their teaching methods and curriculum design to enhance the effectiveness of medical education.

The metaverse platform offers research opportunities in the field of medical education. Researchers can leverage this platform to explore innovative teaching methods and strategies.^5^ Through the design of experiments and surveys, researchers can observe students’ learning behaviors and outcomes within virtual medical environments, analyze the collected data, and draw meaningful conclusions. These studies contribute to uncovering effective practices in medical education, providing valuable guidance for educational reform. For instance, we can examine the impact of different teaching methods on student learning outcomes and explore the comparative advantages of VR and traditional instruction. The findings from such studies serve as a scientific foundation for the educational community, facilitating the continuous enhancement of medical education.

However, data analysis and research also encounter certain challenges. First and foremost, ensuring data privacy and security is of utmost importance.^10^^,^^46^ When collecting student data, it is crucial to establish legitimate, transparent, and secure mechanisms for data usage. This necessitates the implementation of a robust data management system that safeguards students’ privacy rights. Secondly, proficient knowledge and technical skills in data analysis are imperative for conducting research. Researchers must possess expertise in data analysis methods, along with an understanding of metaverse platforms. Moreover, they should exhibit scientific research literacy to ensure the reliability and validity of their investigations. Lastly, the quality and reliability of the data must be carefully considered, necessitating measures to ensure data accuracy.

## Challenges and considerations

When implementing virtual medical training, it is crucial to address technical requirements and limitations, privacy and security concerns, ethical implications, and social acceptance. We must ensure the stability and reliability of technical equipment and systems, safeguard the privacy and security of students’ and patients’ personal information, and adhere to ethical principles and moral standards to ensure the quality and legitimacy of the training. By devising effective solutions and coping strategies, we should overcome these challenges and promote the development and application of simulated patient training.

### Technical requirements and limitations

In the implementation of metaverse training, careful consideration must be given to the technical requirements and limitations.^47^ First, the utilization of VR technology necessitates high-performance computing devices and graphics processing capabilities to create realistic virtual environments and simulated patients. Secondly, the equipment requirements for students need to be thoroughly evaluated, encompassing hardware and software compatibility. Moreover, the stability and reliability of the technology are essential to ensure system functionality and a seamless user experience.

### Solutions to privacy and security issues

Privacy and security issues hold great significance in virtual training endeavors.^7^ Firstly, it is imperative to safeguard students’ and patients’ personal information by complying with relevant data protection laws and regulations. Secondly, robust data management and protection mechanisms, including data encryption and secure transmission, must be established to prevent data breaches and misuse. Additionally, clear privacy policies and usage agreements should be in place to inform users about data collection and usage practices and obtain explicit consent.

### Ethical implications

Ethical implications within simulated patient training necessitate careful consideration and proactive measures.^48^ First, informed consent from students and patients must be ensured by providing clear information regarding the purpose, process, and potential risks associated with their participation, while respecting their autonomy. Second, an ethical review mechanism should be established to scrutinize the design and implementation of simulated patient training, ensuring compliance with ethical principles and moral standards. Additionally, the establishment of reporting and complaint mechanisms allows students and patients to raise concerns and provide feedback in a timely manner, promoting the quality and transparency of the training.

### Social acceptance and promotion of the application

As an emerging educational approach, metaverse training requires societal acceptance and recognition.^10^^,^^49^ To achieve this, enhanced publicity and promotion efforts are necessary to increase public awareness and understanding of simulated patient training. Collaboration with relevant institutions and organizations is also essential in order to foster the application and development of simulated patient training. By enhancing social acceptance and promoting application, we may broaden the reach of the training and enhance its impact and effectiveness.

## Case studies of successful metaverse implementation

To ensure methodological consistency and to move beyond narrative case descriptions, the three case studies cited in this section were synthesized into a structured evidence summary (Table 1). The table presents the learning context, participants, technological platforms, specific metaverse features, learning outcomes, and methodological quality of each study. This overview provides a transparent basis for the subsequent case-by-case discussion.

**Table 1:** Evidence summary of studies on immersive/metaverse-enabled medical education

Study (Author, Year)	Setting	Participants	Technology/Platform	Metaverse Features	Key Outcomes	Study Quality
**Chen**et al. **(2024)** – *BMC Medical Education*	China; tertiary hospital; laparoscopic training	27 OR nurses, 31 postgraduates, 16 residents	VR laparoscopic simulator with haptic feedback (Camera navigation, Peg transfer, Coordination, Fine dissection)	single-user immersive VR; no persistence or identity continuity	significant improvement in all four modules (*p* < 0.001); post-training group differences eliminated	before–after design; transparent outcomes; moderate quality
**Watari**et al. **(2020)** – *IJERPH*	Japan; pre-clinical clerkship lecture hall	169 fourth-year medical students	virtual patient simulation (*Body Interact®*) with two acute care scenarios	partial metaverse traits (multi-user synchronous, no persistence/interoperability)	mean total score 10.1 → 13.0; clinical-reasoning median 5 → 8 (*p* < 0.001); modest knowledge gain	single-center pre-post; valid statistics; short-term outcome focus; moderate quality
**Zhou**et al. **(2022)** – *LNDECT/Springer*	China: first-year experimental course	two parallel classes (VR vs. control)	VR simulation laboratory platform (first-level VR system)	single-user VR; no multi-user or persistent environment	higher course satisfaction (68% vs. 40%) and exam gains (82.31 vs. 77.68) in VR group	quasi-experimental; questionnaire + exam data; moderate quality

*Note*: “Metaverse features” were assessed against the operational definition (shared, persistent, multi-user, interoperable). Studies lacking persistence or identity continuity were coded as immersive VR rather than full metaverse environments.

As summarized in Table 1, while all three studies demonstrate measurable improvements in learners’ engagement or skills, only one exhibits partial metaverse characteristics. Most implementations remain at the immersive VR stage, underscoring the gap between isolated simulation and truly networked, persistent learning environments.

To further clarify the strength and scope of the learning outcomes evidenced in these studies, we interpreted the results through Kirkpatrick’s four-level evaluation framework—reaction, learning, behavior, and results.^50^

Most immersive or metaverse-enabled interventions correspond to Levels 1–2, reflecting learners’ positive reactions (e.g., satisfaction and engagement) and measurable improvements in knowledge or skills. For instance, Chen et al. (2024) and Zhou et al. (2022) both demonstrate Level 2 learning gains, while Watari et al. (2020) additionally capture short-term behavioral change in clinical reasoning (level 3). None of the reviewed studies yet reach Level 4, which concerns long-term organizational or clinical outcomes.

This mapping indicates that current metaverse-based medical-education initiatives remain largely at the formative stages of evaluation and underscores the need for future research to extend assessment toward sustained behavioral and institutional impact.

### Virtual surgical training

VR technology offers medical students the opportunity to engage in surgical simulations within virtual environments, enhancing their operational skills. The virtual surgical training case study aimed to evaluate the impact of virtual surgical simulation training on the laparoscopic skills of operating room nurses, graduate clinical medical students, and residents.^51^ Additionally, the study sought to compare the differences in skill development between these groups before and after the training. The study was composed of 27 operating room nurses, 31 graduate students in clinical medicine, and 16 residents from a tertiary care hospital in China. Employing a self-controlled research design, participants completed tasks within four training modules, evaluated over a six-week period of continuous laparoscopic simulation training. The findings revealed significant enhancement in laparoscopic skills across all participant groups following the completion of virtual surgical simulation training. This real-world application showcases the potential of metaverse technology in medical education, enabling students to gain practical experience and fostering skill improvement.

### Virtual patient simulation

Virtual patient simulation training offers medical students the opportunity to enhance their diagnostic and therapeutic skills by engaging in simulations of clinical scenarios within a virtual environment that closely resembles real-life situations. The study provides valuable insights into the effectiveness of virtual patient simulation training.^52^

The study involved 210 fourth-year medical students who participated in a 2-h virtual patient simulation session. Learning outcomes were assessed through a pre and post-session multiple-choice questionnaire. The results demonstrated a significant improvement in participants’ test scores on knowledge items. The study suggests that virtual patient simulation is more effective in enhancing medical students’ clinical reasoning skills compared to traditional knowledge-based instruction. Therefore, the widespread adoption of virtual patient simulation software programs can optimize the effectiveness of medical school curricula.

### Virtual laboratory

The utilization of VR technology allows medical students to engage in experimental operations within virtual laboratories, thereby enhancing their practical skills and scientific research capabilities. The study conducted provides valuable evidence supporting the implementation of virtual laboratory cases.^53^

According to their research, the application of VR technology in medical laboratory operations has the potential to improve medical students’ practical skills and scientific research abilities. The study selected first-year medical students from a university as participants, with the first class serving as the experimental group, where a medical laboratory course was conducted using the VR system. The second class served as the control group and received traditional multimedia courseware instruction. The experimental and control groups were parallel groups, with similar average grades and intellectual abilities among the students. In the pretest, there was no significant difference in the average test scores between the two groups. However, in the post-test, the experimental group exhibited a significant increase of more than 5 points in their average academic performance compared to the control group, indicating a notable teaching effect. Through interviews with students from the experimental group, it was found that the VR system was preferred over other teaching methods for medical experiments. It offered an immersive learning experience, increased interest in medical experiments, and facilitated a better understanding of medical experimental knowledge through image processing.

### Characteristics and lessons learned from successful cases

Case studies on virtual surgery training, virtual patient simulation training, and virtual laboratories demonstrate the potential of metaverse technology in medical education and its positive impact on skill enhancement. By analyzing these cases, we offer valuable insights for future metaverse applications, further advancing the development and utilization of metaverse technology in medical education. These metaverse application cases exhibit the following characteristics:

The utilization of advanced technologies, such as VR and AR, in medical education can provide students with an immersive learning experience that facilitates better comprehension and application of knowledge. By creating realistic virtual environments, VR and AR enable students to engage in hands-on learning, practice clinical skills, and make informed decisions in simulated real-life scenarios.

Clear educational objectives and tailored designs based on actual needs are crucial in ensuring alignment with the requirements of medical education. By identifying specific learning outcomes and designing educational content accordingly, educators can optimize the learning experience and enhance student understanding and retention of medical knowledge.

Emphasizing student engagement is essential in medical education. Interactive and personalized learning experiences foster student interest and motivation, leading to improved learning outcomes. By incorporating interactive elements, such as gamification, simulations, and case-based learning, educators can create a dynamic and engaging learning environment that promotes active participation and critical thinking.

The integration of metaverse technology with real-world medical education scenarios further enhances the learning experience. By simulating medical situations in virtual environments that closely resemble real-life settings, students can practice clinical skills, make diagnostic decisions, and experience the consequences of their actions. This practical and pragmatic approach enables students to develop and refine their clinical skills and decision-making abilities in a safe and controlled environment.

The establishment of an assessment and feedback mechanism to track students’ learning progress and provide guidance for improvement through measurement and feedback. This assessment and feedback mechanism facilitates continuous optimization of teaching methods and enhances student learning outcomes.

### Quantitative substantiation of educational effectiveness

To strengthen the evidence base beyond descriptive observations, quantitative results from the three core cases were emphasized. Across these studies, learning outcomes consistently showed statistically significant improvements, such as increased procedural accuracy (*p* < 0.001) in Chen et al. (2024), higher clinical-reasoning scores (*p* < 0.001) in Watari et al. (2020), and enhanced examination performance in Zhou et al. (2022). Together, these findings provide concrete numerical support that immersive and metaverse-enabled learning environments yield measurable gains in knowledge and skills, thereby substantiating the educational effectiveness discussed in this review.

Recent systematic and scoping reviews published between 2023 and 2025 (e.g., Ghaempanah et al., 2024; Popov et al., 2024; Fajnerova et al., 2024; Chaddad & Jiang, 2025; Wickramasinghe & Vincent, 2025) have primarily focused on isolated immersive technologies such as virtual or AR and their short-term educational effects. While these reviews have advanced understanding of immersive learning in medicine, they have not integrated these findings within a unified metaverse framework or systematically compared evidence based on metaverse-specific features. The present study extends this literature by combining a taxonomy-based conceptual distinction among VR, AR, MR, and the metaverse with a PRISMA-ScR synthesis and structured evidence table linking metaverse characteristics to learning outcomes and study quality. These analytical and methodological advances clarify what this paper uniquely contributes to the evolving field of metaverse-enabled medical education.

Comparatively, current evidence converges on the short-term benefits of immersive learning while diverging in long-term outcome evaluation and ethical implementation. This review, therefore, bridges prior fragmented findings by positioning the metaverse as an integrative framework connecting these disparate results.

## Future prospects and importance of the metaverse in advancing medical education

Future empirical investigations could adopt mixed-methods or longitudinal designs to quantify performance improvements and user adaptation across different metaverse platforms. The metaverse holds the potential to advance medical education. Firstly, through immersive learning experiences, the metaverse enables medical students to simulate real clinical scenarios in virtual environments, thereby enhancing their skills and decision-making abilities. Virtual surgery training, utilizing VR technology, enables students to improve their surgical skills through realistic surgical simulations. Similarly, patient simulation training allows students to practice diagnosing and treating patients in virtual clinical scenarios, leading to improved clinical competence.

Metaverse applications address the limitations of practical training and experimentation in medical education. Virtual laboratories, for instance, provide students with opportunities to conduct experimental procedures in a controlled virtual environment, thereby enhancing their experimental skills. This virtual laboratory environment offers ample practice opportunities and reduces the risks associated with physical experimentation.

Furthermore, the metaverse facilitates interdisciplinary collaboration and distance learning. Through virtual teamwork and remote collaboration, medical students engage in real-time interactions with professionals from diverse fields, fostering interdisciplinary collaboration. This collaboration promotes knowledge exchange and collaborative innovation across different domains.

Moreover, the metaverse brings forth opportunities for innovation and personalized learning in medical education. Students can tailor their learning experiences according to their individual needs, selecting learning paths and content that suit them best. This personalized approach to learning enhances students’ learning satisfaction.

Additionally, the metaverse promotes the sharing and globalization of medical education resources. Leveraging virtual platforms and network technologies, medical education resources transcend the constraints of time and space, enabling the sharing of educational resources on a global scale. Medical students access high-quality educational resources from around the world, thereby fostering the global development of medical education.

However, the practical implementation of metaverse-based medical education requires careful consideration of feasibility and readiness. Although the pedagogical potential is clear, large-scale adoption depends on several enabling conditions. Feasibility is often constrained by pilot-scale deployment, limited curricular integration, and uneven technological access across institutions. Cost remains a major barrier, encompassing initial hardware investment, software licensing, and ongoing maintenance of digital platforms. Adequate technical infrastructure, including stable connectivity, secure servers, and interoperability standards, is essential to ensure seamless multi-user interaction and protect learner data. Faculty development is equally critical, as instructors must be trained not only in operating immersive systems but also in facilitating virtual instruction and assessment. Finally, institutional readiness, spanning administrative support, ethical governance, and policy alignment, is required to sustain long-term implementation. Addressing these dimensions will transform the metaverse from a promising technological experiment into a scalable and sustainable component of medical education.

Beyond pedagogical and technical challenges, the metaverse introduces distinct ethical and governance concerns that must be proactively addressed. Data protection is critical, as virtual learning environments collect biometric, behavioral, and interaction data that require secure storage and compliance with privacy regulations such as GDPR and HIPAA. Avatar professionalism raises questions of identity representation, conduct, and accountability—medical learners and instructors should maintain the same professional standards in virtual spaces as in real clinical settings. Robust user authentication mechanisms are necessary to verify participants’ identities, prevent impersonation, and ensure equitable participation in assessments. Accessibility must also be prioritized; institutions should ensure that metaverse platforms accommodate users with disabilities and avoid reinforcing digital divides based on hardware cost or network access. Finally, intellectual property management requires clear institutional policies defining ownership of virtual assets, educational content, and collaboratively created simulations. Establishing governance frameworks across these dimensions is essential to ensure ethical integrity, legal compliance, and equitable participation in metaverse-enabled medical education.

## Conclusion

In conclusion, the literature review has shed light on the relationship between metaverse technologies and medical education, as well as how the characteristics of the metaverse align with the needs of medical education. The review revealed that simulated patient training, simulation training, collaborative learning, cross-location learning, and data analytics were extensively discussed in terms of the advantages and opportunities offered by the integration of the metaverse in medical education. Additionally, we addressed challenges and considerations such as technical requirements and limitations, privacy and security concerns, ethical implications, coping strategies, social acceptance, and the diffusion of metaverse applications. Notably, successful implementation case studies, including virtual surgery training, simulated patient training, and virtual laboratories, have demonstrated the practical applications of the metaverse in medical education. Looking forward, the metaverse holds promising prospects and significance in advancing medical education. It has the potential to revolutionize traditional medical education by providing more practical learning experiences, facilitating collaborative learning, and enabling the global sharing of educational resources. These findings offer valuable insights into the application and development of the metaverse in medical education, providing a foundation for future research and practical implementation.

The practical implications of the metaverse in medical education are highly significant. It offers immersive and practical opportunities for students to simulate real medical scenarios in virtual environments, enabling them to engage in actual operations and decision-making. This immersive experience enhances students’ practical skills and their ability to handle complex situations. Moreover, the metaverse facilitates interdisciplinary collaboration, allowing medical education to intersect with fields such as computer science and human-computer interaction. This collaboration fosters innovative teaching methods and tools, leading to a more comprehensive and integrated medical education that cultivates students’ integrative skills and teamwork. Furthermore, the metaverse enables personalized learning by providing customized content and learning paths tailored to students’ individual needs and interests. This personalized approach stimulates students’ motivation and engagement, resulting in improved learning outcomes.

The application of the metaverse also holds theoretical significance for medical education. It opens up new research fields and perspectives, promoting the development and innovation of educational theory. Additionally, the metaverse offers new research objects and methods for disciplines such as pedagogy and psychology, encouraging interdisciplinary collaboration and knowledge sharing. Therefore, the metaverse has profound practical and theoretical implications in medical education. It provides a diverse learning environment and teaching tools, enhances students’ practical abilities and overall qualities, promotes educational reform and innovation, and inspires new directions for educational research and theory development.

### Limitations of the study

There are still certain limitations in the current research on the application of the metaverse in medical education, necessitating further in-depth exploration and resolution. More empirical studies are required to assess the actual effects and learning outcomes of the metaverse in medical education. Long-term research and empirical validation are necessary to fully comprehend the specific impact of the metaverse on student learning and teaching outcomes. The implementation and application of metaverse technology in medical education also encounter various technical, educational, and social challenges. Enhancing the performance and usability of the metaverse platform and addressing issues such as network latency and bandwidth limitations is crucial. Additionally, the development of teaching scenarios and methods tailored to diverse learning objectives and needs is imperative.

Looking ahead, we offer suggestions for future research. On the technical front, we recommend continuous research and development of metaverse technologies to enhance their performance and usability, and address privacy and security concerns. This will ensure a favorable user experience and guarantee security in the metaverse environment. In terms of education, we advocate for more empirical studies to evaluate the actual effects of the metaverse in medical education, as well as explore additional teaching scenarios. This will provide a better understanding of the effectiveness of metaverse applications in medical education and offer guidance and support to educators. Furthermore, we encourage greater interdisciplinary collaboration and foster cooperation between medical education and fields such as computer science. Through collaborative efforts and knowledge exchange, we can better promote the application and development of metaverse technologies in medical education, ultimately providing enhanced learning experiences and educational opportunities for students.

## Data and code availability

This study is a scoping review and did not generate new data or code. All data analyzed in this review were obtained from previously published studies that are publicly available through academic databases. No datasets or software were created by the authors for this work.

## Acknowledgments

No funding was received for conducting this study.

## Author contributions

Conceptualization, methodology, investigation, data curation, visualization, writing – original draft, and writing – review and editing: Z.C.

## Declaration of interests

The authors declare no competing interests.
